# The Cellular Prion Protein PrP^c^ Is Involved in the Proliferation of Epithelial Cells and in the Distribution of Junction-Associated Proteins

**DOI:** 10.1371/journal.pone.0003000

**Published:** 2008-08-20

**Authors:** Etienne Morel, Stéphane Fouquet, Carine Strup-Perrot, Cathy Pichol Thievend, Constance Petit, Damarys Loew, Anne-Marie Faussat, Lucile Yvernault, Martine Pinçon-Raymond, Jean Chambaz, Monique Rousset, Sophie Thenet, Caroline Clair

**Affiliations:** 1 Centre de Recherche des Cordeliers, Université Pierre et Marie Curie-Paris 6, UMR S 872, Paris, F-75006 France; 2 INSERM, U 872, Paris, F-75006 France; 3 Université Paris Descartes-Paris 5, UMR S 872, Paris, F-75006 France; 4 Radiosensibilité des tissus sains, UPRES EA 27.10, Institut Gustave Roussy PRI, Villejuif F-94805, France; 5 Laboratoire de Spectrométrie de Masse Protéomique, Institut Curie, Pavillon Pasteur, 75248 Paris, France; University of Birmingham, United Kingdom

## Abstract

**Background:**

The physiological function of the ubiquitous cellular prion protein, PrP^c^, is still under debate. It was essentially studied in nervous system, but poorly investigated in epithelial cells. We previously reported that PrP^c^ is targeted to cell–cell junctions of polarized epithelial cells, where it interacts with c-Src.

**Methodology/Findings:**

We show here that, in cultured human enterocytes and in intestine *in vivo*, the mature PrP^c^ is differentially targeted either to the nucleus in dividing cells or to cell–cell contacts in polarized/differentiated cells. By proteomic analysis, we demonstrate that the junctional PrP^c^ interacts with cytoskeleton-associated proteins, such as gamma- and beta-actin, alpha-spectrin, annexin A2, and with the desmosome-associated proteins desmoglein, plakoglobin and desmoplakin. In addition, co-immunoprecipitation experiments revealed complexes associating PrP^c^, desmoglein and c-Src in raft domains. Through siRNA strategy, we show that PrP^c^ is necessary to complete the process of epithelial cell proliferation and for the sub-cellular distribution of proteins involved in cell architecture and junctions. Moreover, analysis of the architecture of the intestinal epithelium of PrP^c^ knock-out mice revealed a net decrease in the size of desmosomal junctions and, without change in the amount of BrdU incorporation, a shortening of the length of intestinal villi.

**Conclusions/Significance:**

From these results, PrP^c^ could be considered as a new partner involved in the balance between proliferation and polarization/differentiation in epithelial cells.

## Introduction

The cellular prion protein (PrP^c^) is a ubiquitous glycoprotein anchored to the outer leaflet of the plasma membrane, in raft domains, through a glycosylphosphatidylinositol (GPI) moiety [1]. Its central role in transmissible spongiform encephalopathies has been clearly demonstrated for many years [2]–[4] and efforts have been made to determine its biological role apart from pathological situations. Although many cells and tissues, such as blood lymphocytes, muscle, heart, kidney, digestive tract and skin, express PrP^c^ [5]–[9], most of the studies concerning its physiological function have been performed on nerve cells. In these models, it has been established that PrP^c^ binds copper [10] and can homodimerize [11] or interact with other proteins, among which are synapsin Ib, Grb2, Pint1, LRP/LR, and N-CAMs [12]–[19]. It has also been reported that PrP^c^, via interaction with phosphorylated Fyn [20], participates in cell redox homeostasis through ROS production [21]. In addition, it has been shown that multiple biochemical changes occur in prion protein knockout mice. They include increased levels of NF-κB and COX-IV and decreased levels of p53 and Cu, Zn superoxide dismutase activity, along with an increased neuronal sensitivity to oxidative stress in cultured cells isolated from these mice [22].

Much less is known about the role of PrP^c^ in extra-neuronal tissues. In epithelial cells, PrP^c^ was reported to be directed to basolateral membranes of MDCK and FRT epithelial cells [23]. We have shown that PrP^c^ is expressed in enterocytes [24], which are highly polarized epithelial cells of the intestinal epithelium. Interestingly, we showed that, in polarized/differentiated enterocytes, PrP^c^ is targeted to the lateral junctional complexes of adjacent cells where it interacts with Src kinase [24]. This tyrosine kinase is known targeted to cell–cell junctions where it phosphorylates substrates that induce adhesion turnover and actin remodeling [25]. Such a localization of PrP^c^, was also observed in human keratinocytes [24] and in endothelial cells [26], opening questions about the role of PrP^c^ in cell–cell junctions of physiological barriers.

To address this question, we focused our study on intestinal epithelium and on enterocytes, the major cell population of this epithelium. Intestinal epithelium undergoes a rapid renewal throughout life (for review see [27]). Such a process requires a continuous coordination between proliferation, differentiation and death programs, along with a remodeling of cell-matrix and cell–cell contacts responsible for cell polarization. In crypts are localized stem cells and dividing cells, which migrate up to the villus where differentiation takes place. In the present work, we have analyzed whether the sub-cellular localization of PrP^c^ varied in relation with cell proliferation, cell polarization and the state of cell–cell junctions in human intestinal epithelium *in vivo* and in the human Caco-2/TC7 enterocytes [28], which reproduce in culture the sequence of division and polarization/differentiation. In this enterocyte model, we characterized the partners of PrP^c^ in cell–cell junctions. Finally, the impact of the invalidation of PrP^c^ on the distribution of cell–cell junctions-associated proteins, the process of cell proliferation and the morphology of the intestinal epithelium was analyzed.

## Materials and Methods

### Reagents

Except when indicated, all chemicals were purchased from Sigma (Sigma-Aldrich, Saint Quentin Fallavier, France). Mouse 12F10 (against peptide 142–160) and SAF32 (against peptide 79–92) anti-human PrP^c^ monoclonal antibodies were obtained from S.P.I BIO (Montigny le Bretonneux, France). Rabbit anti-human Ki67 polyclonal antibody and rat anti-mouse E-cadherin monoclonal antibody (ECCD-2) were purchased from Zymed Laboratories (San Francisco, CA). Rabbit anti-human Src (sc-18), and goat anti-human poly (ADP-ribose) polymerase (PARP) antibodies were purchased from Santa Cruz Biotechnology (Santa Cruz, CA). Mouse anti-human calnexin, desmoglein, plakoglobin and annexin A2 monoclonal antibodies were purchased from BD Biosciences (Erembodegem, Belgium). Rabbit anti-human PrP^c^ (Ab 703), anti-pan desmoglein, anti-desmoplakin, anti phospho-S10-Histone H3 (Ab5176) polyclonal antibodies and rat anti-BrdU monoclonal antibody were purchased from Abcam (Cambridge, UK). Secondary CY2-, CY3- and CY5-labelled antibodies were purchased from Jackson Immuno-Research (West Grove, PA). F-actin was labelled with phalloidin-FITC. Endoglycosidase F was purchased from VWR (Fontenay sous bois, France). The biotinylated pro-aerolysin bacterial toxin [29] was a kind gift from Gisou van der Goot (Ecole Polytechnique de Lausanne, CH-1015 Lausanne, Switzerland).

### Cell culture

All culture media were purchased from Gibco, Invitrogen Life Technologies (Cergy Pontoise, France). Caco-2/TC7 cells [28] were cultured with high glucose DMEM (Dulbecco's modified Eagle's medium) Glutamax I supplemented with 20% heat inactivated (56°C, 30 min) fetal calf serum (AbCys, Paris, France), 1% non-essential amino acids, penicillin (100 IU/ml) and streptomycin (10 µg/ml) in a 10% CO_2_/air atmosphere. The medium was changed every day. Depending on experiments, cells were plated on 1 µm pore size microporous PET filters (Falcon, BD Biosciences, Franklin lakes, NJ), or in plastic flasks (Falcon) or on glass lamellae (Polylabo, Strasbourg, France).

### Cells treatments

#### Cycloheximide treatment

When indicated, the cells were treated with cycloheximide (10 µM final concentration).

#### siRNA transfection

siRNA corresponding to the human *Prnp* gene from codon 399 to 417 was synthesized by MWG Biotech (Ebersberg, Germany). The specific human siRNA sequence used was: 5′-GCC-GAG-UAA-GCC-AAA-AAC-CTT-3′ (sense). Cells were seeded at 5000 cell/cm^2^ on plastic or glass lamellae. siRNAs were mixed with Oligofectamine reagent (Invitrogen Life Technologies) for 15 min and Opti-MEM medium without serum was added according to the manufacturer's instructions. The final concentration of siRNA was 400 nM. After incubation for 6 hours at 37°C, Opti-MEM supplemented with 60% fetal calf serum was added to reach a final 20% serum concentration. A mouse PrP^c^ siRNA sequence (5′-GCC-CAG-CAA-ACC-AAA-AAC-CTT-3′), inefficient on human PrP^c^ RNA, and a scramble siRNA (5′-CCG-AGA-AGU-AAA-GCC-AAC-CTT-3′) were used as controls along with cells incubated with Oligofectamine reagent only. After 24 h, the medium was changed for the standard medium and the cells from all conditions were maintained in culture for the indicated times, the medium being changed every day.

### GFP-PrP^c^ plasmid construct and cell transfection

pEGFP-mouse PrP^c^ plasmid [30] was obtained from MA Prado. GFP-PrP^c^ was originally under the control of CMV promoter in this plasmid. To allow the expression of GFP-PrP^c^ in differentiated cells, the corresponding sequence was subcloned into pGL2basic vector (Promega France) under the control of SV40 promoter. Caco-2/TC7 cells were transfected on day 2 with oligofectamine (Invitrogen, France) according to the manufacturer's instructions. After selection with G418 (Gibco BRL, France), transformed cells were allowed to expand prior to sorting, on the basis of GFP fluorescence, in an ALTRA cell sorter (Beckman Coulter, France).

### Immunofluorescence analysis

Cells were washed twice with PBS containing 1 mM CaCl_2_ and 0.5 mM MgCl_2_, and fixed for 30 min with 4% paraformaldehyde (wt/vol) in PBS at 4°C. After an extensive washing in 150 mM glycine in PBS (PBS-glycine), the cells were permeabilized by incubation for 30 min in 0.1% Triton X-100 in PBS and washed in PBS-glycine followed by PBS plus 1% BSA. Cells were incubated for 60 min at room temperature with primary antibodies in PBS supplemented with 1% BSA, washed with PBS and stained with secondary antibodies in PBS with 1% BSA for 60 min at room temperature in the dark. After extensive washing in PBS, cells were mounted in Fluoprep (BioMérieux, Marcy l'Etoile, France), and examined by epifluorescence microscopy (Axiophot microscope connected to Axiocam camera using Axiovision 4.5 software; Carl Zeiss). Confocal microscopy (LSM 510 microscope; Carl Zeiss, Jena, Germany) was used for the observation of cells cultured on microporous filters, which are strongly autofluorescent and generate excessive background in epifluorescence. X–Z planes resulted from 0.1 µm stacks.

### Tissue analysis

PrP^c^ knock out mice [2] backcrossed on C57Bl6 and their wild type C57Bl6 counterparts were purchased from CDTA (Orléans, France). After removal of intestine in wild type or PrP^c^ knock out mice, 2 cm segments of duodenum and jejunum were cut, gently flushed with PBS and opened longitudinally. Rolled segments were frozen in cryo-embedding media (OCT) and stored at −80°C until cryostat sectioning (10–20 µm). Sections were applied onto gelatine-coated glass slides, fixed in paraformaldehyde solution (4%), permeabilized with Triton before DAPI staining and then mounted in fluoprep solution.

Mitotic crypt cells were labeled with an anti-phosphoS10-Histone H3, using the same protocol as for cell immuno-labeling. To label proliferating intestinal crypt cells in S-phase, PrP^c^ knock out and wild-type C57Bl6 mice were given an intraperitoneal injection of 5-bromo-2′-deoxyuridine (BrdU; Sigma; 120 mg/kg body weight) 90 min before sacrifice. Paraffin sections of alcohol-formalin-acetic acid (AFA)-fixed jejunum were incubated for 30 min in 2.5 N HCl before processing for immunofluorescent labeling with anti-BrdU antibody.

Sections from paraffin-embedded human jejunum were sequentially treated with xylene (2×5 min), 100% EtOH (2×5 min), 95% EtOH (1×5 min) and then rinsed with water. Antigens retrieval was performed by boiling slides in 10 mM citrate buffer (pH 6) for 10 min. After washes in PBS, sections were fixed and then the same protocol as described above for immunofluorescence was used.

### Immunoelectron microscopy

Caco-2/TC7 cells, grown on Thermanox coverslips (Agar scientific), were fixed with acetone. After incubation with 12F10 monoclonal antibody, gold (1 nm particles)-labelled goat anti mouse IgG (Amersham Biosciences) were used as secondary antibodies and the resulting signal was enhanced by the Intense TM M silver enhancement kit (Amersham Biosciences). After alcohol-graded dehydratation, sections were embedded in Epon and ultrathin sections were analyzed (Jeol 100 CX II). For desmosomal structure analyses, intestine was flushed with cold 0.1 M Phosphate buffer (pH 7.4), ligatured and filled with cold 2.5% glutaraldhehyde/0.5% tannic acid in 0.1M cacodylate buffer at pH 7.4 for 2H. Fine samples of intestine were cut and fixed by 0.6% glutaraldhehyde/0.5% tannic, which stains and preserve the ultrastructure of phospholipids [31] in the same buffer overnight at 4°C. Postfixation was carried out in 2% osmic acid in 0.1 M Phosphate buffer for 1h at 4°C. Samples were then dehydrated in graded alcohol and embedded in Epon resin (Poly/Bed 812, Polysciences Inc.Warrington, PA). Ultrathin sections of around 65 nm were counterstained with uranyl acetate (30 min, 40°C) and lead citrate (10 min, 25°C) using an LKB 2168 ultrostainer. Observations were made using a JEOL CX100 equipped with a Gatan Digital camera (3.11.0) and the migrographs were processed with Gatan software.

### Sub-cellular fractionation

#### Preparation of detergent-insoluble membranes on sucrose gradient

Caco-2/TC7 cells were homogenized on ice for 30 min in 2 ml of 10 mM Tris-HCl pH 8, 150 mM NaCl buffer (TN) containing 1% Triton X100 or in 2 ml of 20 mM Tris-HCl pH 7.8, 250 mM sucrose, 1 mM CaCl_2_ and 1 mM MgCl_2_ without detergent [32]. Anti-protease cocktail and anti-phosphatases (orthovanadate and beta-glycerophosphate) were added in both conditions. The cell homogenate was then adjusted to 40% sucrose by addition of 2 ml sucrose (80% in TN). The resulting 4 ml were covered with 4 ml of 30% sucrose and 4 ml of 5% sucrose and centrifuged for 16 h (39,000 rpm, 4°C) in a SW-41 rotor (Beckman Instruments, Gagny, France). Sequential 1 ml fractions were then collected from the top of the tube and the turbid fraction containing the floating detergent-insoluble membranes (fraction 4) was adjusted to 11 ml in TN buffer and centrifuged in a SW-41 rotor (35000 rpm, 1h). The pellet was dissolved in TN buffer containing 1% NP40, anti-proteases and anti-phosphatases and stored at −80°C until immunoprecipitations.

#### Nuclei and crude membrane preparations

Proliferating or polarized/differentiated Caco-2/TC7 cells were washed twice in 10 mM Tris-HCL pH 7.5 containing 20 mM KCl, 2 mM CaCl2, 2 mM MgCl2 and 0.2 mM spermidine (TKCM buffer) and scrapped in TKCM containing 1% Triton X-100, 1 mM phenylmethylsulfonylfluoride (PMSF), anti-proteases and anti-phosphatases. Nuclei were pelleted by centrifugation at 1000g for 10 min at 4°C and supernatants corresponding to cytosolic and membrane proteins were stored at –80°C until analysis. The pellets were washed in TKCM buffer and nuclear proteins were extracted with 2M NaCl in TKCM buffer for 1h at 4°C. Excess NaCl was removed by overnight dialysis against PBS at 4°C.

### Immunoprecipitation and immunoblotting analyses

The raft fractions were immunoprecipitated with rabbit anti-PrP^c^ (Ab 703) polyclonal antibodies, or non-specific rabbit immunoglobulins or anti-pan desmoglein antibodies coupled to protein A-sepharose 4B (Amersham Biosciences, Orsay, France). For SDS-PAGE, samples were boiled for 10 min in Laemmli buffer (2.5% SDS final concentration) and fractionated under reducing conditions in polyacrylamide gel. Proteins were transferred onto nitrocellulose membranes (Bio-Rad), blocked 2h with 5% non-fat dried milk in TBS/0.1% Tween 20 (TBST). After two washes in TBST, membranes were incubated (1h at room temperature) with specific antibodies. After three washes in TBST, the membranes were incubated (1h at room temperature) with peroxidase-labelled (HRP) secondary antibodies (Amersham Biosciences) in TBST. After three washes in TBST, bound antibodies were detected by chemiluminescent method (ECL, Amersham Biosciences). The quantitative analyses were performed with a high performance calibrated imaging densitometer (Bio-Rad GS-800) using PD Quest and Image Quant 5.2 softwares.

#### GPI anchor detection

After SDS-PAGE of immunoprecipitated materials and transfer onto nitrocellulose, membranes were incubated two hours in a binding buffer (50 mM NaH2PO4/0.3% Tween 20) before addition of biotinylated pro-aerolysin bacterial toxin. Biotinylated proteins were detected by blotting with HRP-conjugated streptavidin.

#### Endo F treatment

Nuclear proteins and rafts extracts were treated with Endoglycosidase F (10 units/50 µg proteins) in 40 mM sodium phosphate buffer, pH 7.5 containing 0.4% SDS, 20 mM DTT and 0.8% NP40 for 16 hours at 37°C before immunoblot analysis.

### MS analysis

#### SDS/PAGE separation and protein digestion

Raft fractions were immunoprecipitated with anti PrP^c^ antibodies and separated on 4–12% SDS/polyacrylamide gels. After staining with colloidal Commassie blue (G250, Bio-Rad), the visualized bands were cut into slices of 1 mm. Gel slices were then reduced, alkylated and subjected to digestion with trypsin (Roche Diagnostics) as already described [33]. Extracted peptides were dried and solubilized in solvent A (95/5 water/acetonitrile in 0.1% (w/v) formic acid). The total digestion product of a gel slice was used per liquid chromatography-tandem MS (MS/MS) analysis.

#### Liquid chromatography-MS/MS analysis

The extracted peptides were concentrated and separated on a LC-Packing system (Dionex S.A.) coupled to the nano-electrospray II ionisation interface of a QSTAR Pulsar i (Applied Biosystems) using a PicoTip (10 µm i.d., New Objectives, Woburn, MA). The MS/MS data was searched twice by using MASCOT (Matrix Science, London) and PHENYX (Geneva Bioinformatics S.A.) softwares on internal servers, first without taxonomic restriction to reveal the presence of proteins of interest and mammalian contaminants, then again the National Center for Biotechnology Information Human database (National Library of Medicine, Bethesda). All data are manually verified in order to minimise the errors in protein identification and/or characterization.

### Statistical analysis

Statistical analyses were performed using student's *t* test.

## Results

### The cellular prion protein is localized in the nucleus in dividing cells and in cell–cell junctions in polarized epithelial cells

**Figure 1:**
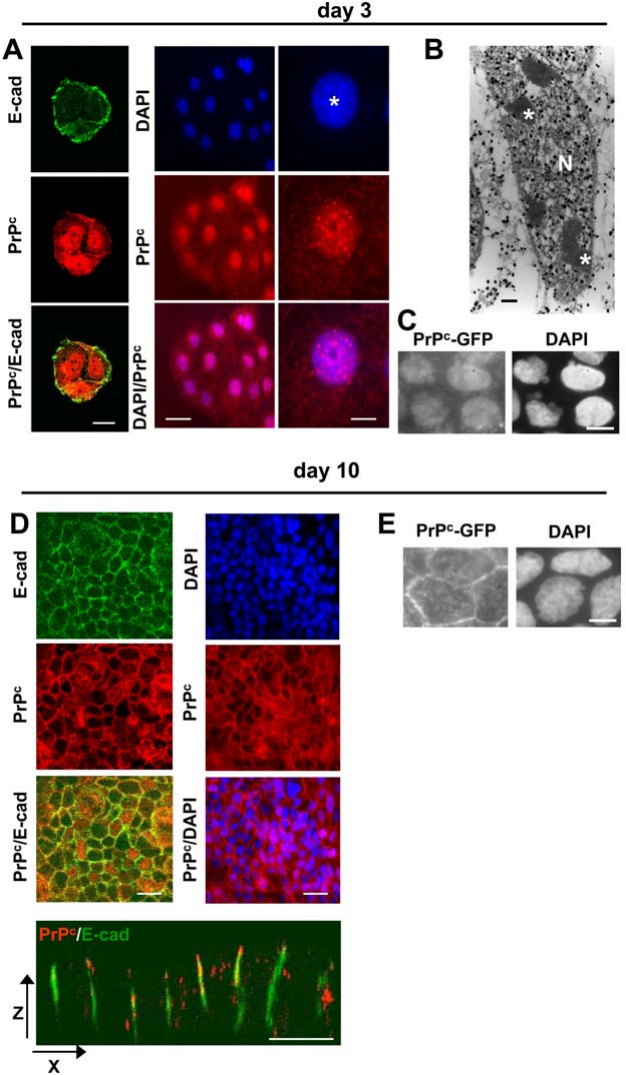
Expression and localization of PrP^c^ in proliferating or differentiated/polarized Caco-2/TC7 cells. Immunofluorescence labeling of PrP^c^ (red, 12F10 antibody) and E-cadherin (green) was performed after 3 (A, left panel) or 10 (D, left panel) days in culture. Nuclei were stained with DAPI (A, middle and right panels, D, right panel). Right panels in A represent an enlargement of a typical nuclear labeling of one cell (_*_, nucleolus). Note that left panels correspond to a cluster of three cells and middle panels to a cluster of 13 cells. (B): Immunoelectron microscopy of PrP^c^ was performed on day 3. (N, nucleus; _*_, nucleolus). (C, E): Sub-cellular localisation of GFP-PrP^c^ was analyzed on day 3 or 10 and compared with DAPI staining (right panels). Note the absence of PrP^c^ and of E-cadherin, used as a marker of the junctional state, at the cell–cell contacts of proliferative cells (A) and their presence in the lateral membranes of polarized/differentiated cells as shown in XY (upper panels D) and XZ (lower panel D) planes. Bars: (A) 10 µm for left panels, 20 µm for middle panels and 4 µm for right panels, (B) 1 µm, (C and E) 10 µm and (D) 20 µm.

We analyzed, by immunofluorescence and immunoelectron microscopy, the distribution of PrP^c^ or of GFP-PrP^c^ in exponentially growing or polarized Caco-2/TC7 enterocytes. Representative images of PrP^c^, E-cadherin and DAPI labeling of the nuclei in exponentially growing Caco-2/TC7 cells (day 3) are shown in Figure 1A. When cells have not yet established well-defined adherens junctions, as shown by the poor expression of E-cadherin at cell–cell contacts (left panel), PrP^c^ was mainly detected intracellularly. Interestingly, this staining co-localized with DAPI labeling, in the nucleus (middle panel). Immunodetected PrP^c^ appeared as dots that were distributed around the nucleolus (right panel). This localization was confirmed by immunoelectron microscopy where the PrP^c^ signal appeared accumulated in the nucleus (Fig. 1B, N) and systematically excluded from the nucleolus (Fig. 1B, _*_). The nuclear localization of the transfected mouse GFP-PrP^c^ at this stage of the culture further strengthened the results obtained for the endogenous protein (Fig. 1C).

In confluent and polarized Caco-2/TC7 cells (day 10), when E-cadherin-dependent junctions were established, PrP^c^ was detected at the lateral membrane, and did not co-localize with DAPI in 90–95% of the cells (Fig. 1D). GFP-PrP^c^ was also found targeted to the lateral membranes of polarized cells (Fig. 1E). This PrP^c^ localization was previously revealed by immunoelectron microscopy [24]. As cells were not synchronized, PrP^c^ was found in the nucleus in the few dividing cells (5–10%) that were still present within the confluent cell layer. In all conditions, a significant proportion of trafficking PrP^c^, which corresponded to approximately 30–40% of the total protein, was also observed in the cytoplasm.

### The cellular prion protein is differently localized in proliferative and differentiated compartments of human intestinal epithelium

**Figure 2:**
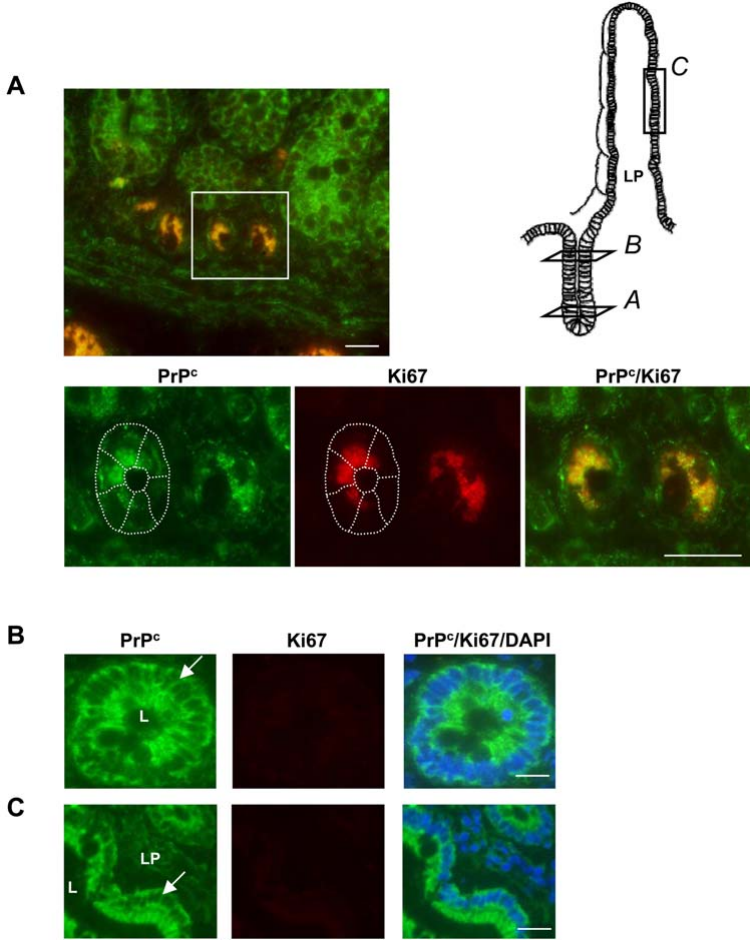
Expression and localization of PrP^c^ and Ki67 in normal human intestinal epithelium. Immunofluorescence labeling of PrP^c^ (green, 12F10 antibody) and Ki67 (red) was performed on thin sections of paraffin-embedded pieces of jejunum. Images in A, B and C correspond to crypts, crypt-villus transition compartment and villus respectively, as indicated on the scheme. Lower panels in A correspond to an enlargement of the crypt zone shown above. In B and C, nuclei were stained with DAPI. Note the colocalization of PrP^c^ and Ki67 in the nuclei of crypt cells and the cytoplasmic and membrane localization of PrP^c^ in Ki67 negative epithelial cells. LP, lamina propria; L, lumen. Arrows point out cell–cell junctions of the epithelium. Bars: 20 µm.

The sub-cellular localization of PrP^c^ was analyzed in human intestinal epithelium and compared in crypts and villi, which correspond to the proliferative and differentiated compartments, respectively. In crypts, PrP^c^ was found to co-localize with Ki-67, a nuclear marker of dividing cells (Fig. 2A). By contrast, in the crypt-villus transition compartment (Fig. 2B) and in villi (Fig. 2C), i.e. as soon as the process of cell division is stopped and the differentiation takes place, the nuclei, visualized by DAPI, were found devoid of Ki67 and of PrP^c^, which was visualized in the cytoplasm and in the lateral membranes of adjacent cells.

### Nuclear and junctional PrP^c^ isoforms exhibit similar post-translational modifications but differ in their stability

**Figure 3:**
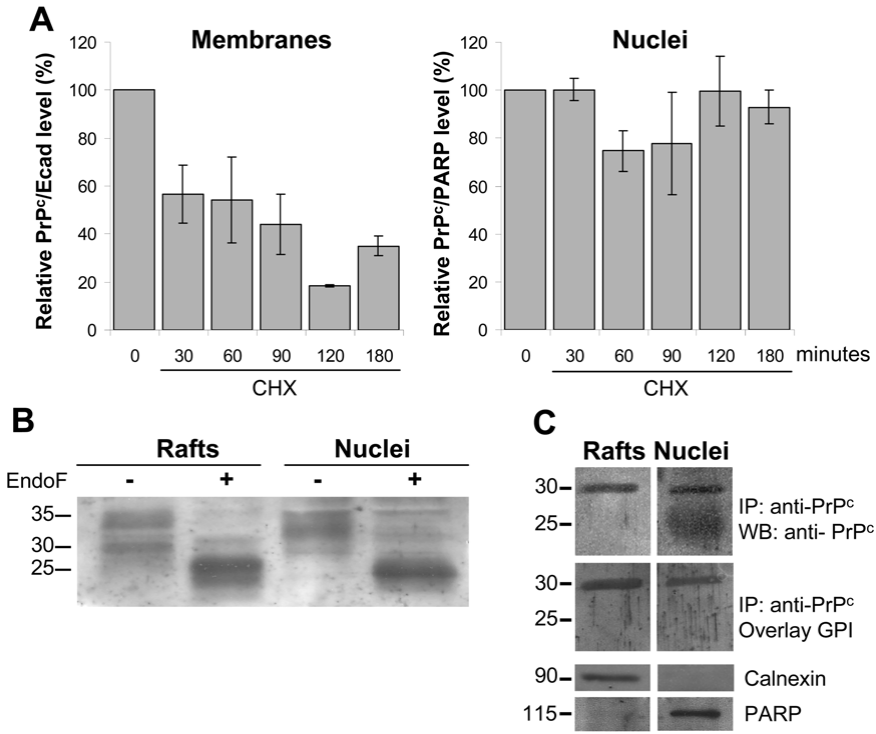
Biochemical characterization of membrane- and nucleus-associated PrP^c^ isoforms. (A): Stability of membrane and nuclear PrP^c^ was analyzed by western blot after treatment of the cells with cycloheximide for the indicated times and purification of membranes and nuclei. Bands obtained in western blots (SAF 32 antibody) were quantified by scanning densitometry. E-cadherin and PARP were used to normalize the values obtained in membrane and nuclear fractions respectively, since both proteins were found stable for the duration of CHX treatment. Histograms correspond to the ratio (%) between PrP^c^ and E-cadherin or PARP from the corresponding scanned bands at each time (mean±SD from 3 independent experiments), the value obtained at time 0 being set at 100. (B): To determine the glycosylation state, rafts and nuclear extracts were treated (+) or not (−) with endo F and PrP^c^ was analyzed by western blot (SAF 32 antibody). Molecular weight in KDa are indicated (C): The presence of a GPI anchor was analyzed after immunoprecipitation of PrP^c^ from rafts or nuclear extracts, SDS-PAGE, transfer and overlay with biotinylated pro-aerolysin bacterial toxin. To check the purity of the extracts, the expression of calnexin (membrane marker) and PARP (nuclear marker) was analyzed by western blot. Molecular weight in KDa are indicated.

The differential sub-cellular localization of PrP^c^ could result from different molecular properties of the protein. Thus, we first compared the stability of PrP^c^ when localized in the nucleus or in the lateral membranes. The amount of PrP^c^ was determined by western blot, in nuclear extracts from proliferative cells (day 3) and membrane PrP^c^-containing raft domains from differentiated Caco-2/TC7 cells (day 10), at different times after inhibiting translation by cycloheximide. Results reported in Figure 3A show that PrP^c^ is much more stable when localized in the nucleus, where degradation could not be observed over a 3 hour period, than at the membrane, where 50% of the protein were degraded at 30 min.

PrP^c^ is submitted to post-translational modifications such as N-linked glycosylation and addition of a GPI anchor. Endo F treatment of nuclear or raft extracts resulted in an equivalent shift of PrP^c^ bands, demonstrating that nuclear and raft PrP^c^ are similarly N-glycosylated (Fig. 3B). Analysis of the presence of a GPI anchor was performed on PrP^c^ immunoprecipitated by the specific polyclonal rabbit antibodies Ab 703, using the biotinylated pro-aerolysin bacterial toxin, which recognizes GPI anchors [29]. A specific band corresponding to the presence of a GPI anchor on PrP^c^ was detected in both nuclear and raft extracts (Fig. 3C, middle panel), at the same molecular weight as the PrP^c^ signal (Fig. 3C upper panel), i.e. at 30 kDa. The absence of contamination of the nuclear and raft extracts with proteins derived from the other compartment was verified. Calnexin protein, which is known to be regularly buoyed with rafts [34] and the poly-(ADP-ribose) polymerase (PARP) protein that is exclusively expressed in the nucleus were used as markers of the respective compartments (Fig. 3C, lower panels).

### Junctional PrP^c^ is part of a complex involving desmosome-associated proteins and c-Src, and interacts with the structural proteins actin and annexin A2

**Figure 4:**
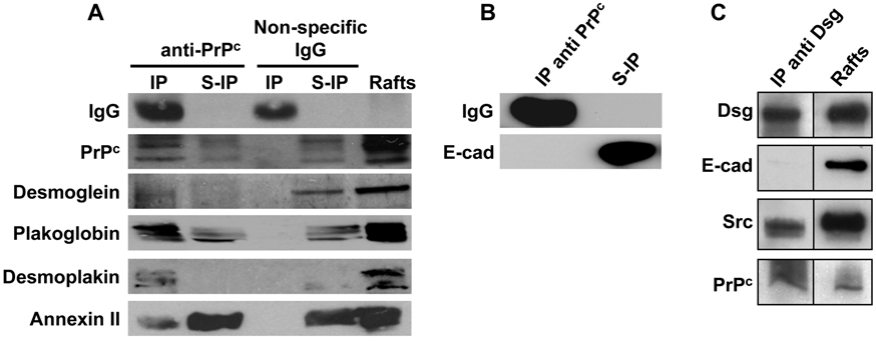
Immunodetection of PrP^c^-associated desmosomal proteins in differentiated Caco-2/TC7 cells. (A): Proteins that were found to interact with PrP^c^ through proteomic study (Table 1) were analyzed by western blot in rafts, and in immunoprecipitated material (IP) or in supernatants of immunoprecipitations (S-IP) from raft extracts of differentiated cells (day 10). Immunoprecipitations were performed with rabbit anti-PrP^c^ antibodies (Ab703) or with non-specific rabbit immunoglobulins as a control. (B): The presence of E-cadherin, which was not identified as a PrP^c^ partner, was checked, after immunoprecipitation with anti-PrP^c^, in immunoprecipitated material (IP) and immunoprecipitation supernatant (S-IP). Note that E-cadherin is only recovered in immunoprecipitation supernatant. (C): desmoglein (Dsg), Src, PrP^c^ and E-cadherin (E-cad) were analyzed by western blot in raft extracts (right lane) or after their immunoprecipitation with anti-desmoglein antibodies (left panel). Note the co-immunoprecipitation of desmoglein, c-Src and PrP^c^ and the absence of E-cadherin co-immunoprecipitation.

A proteomic analysis was undertaken to determine the partners of PrP^c^ in the junctional domains of enterocytes. Results presented in table 1 showed that the membrane PrP^c^ interacts with five desmosome-associated proteins, among which desmoglein, plakoglobin and desmoplakin, and with gamma- and beta-actin and annexin A2, a structural protein that is known to participate in the regulation of actin cytoskeleton dynamics in junctions of epithelial cells [35]. The interaction with the desmosomal proteins and with annexin A2 was corroborated by western blots after purification of rafts in the presence of detergent and immunoprecipitation of PrP^c^ by the specific rabbit polyclonal antibodies Ab 703 (Fig. 4A). Same results were obtained with rafts purified, after sucrose gradients, from cell extracts prepared without detergent (not shown). The absence of interaction between E-cadherin and PrP^c^ [24] was confirmed, since E-cadherin was recovered exclusively in the immunoprecipitation supernatant (Fig. 4B). Interestingly, besides the already reported interaction of c-Src with the junctional PrP^c^ [24], the immunoprecipitation of raft fraction with the anti-transmembrane protein desmoglein antibody revealed a complex including c-Src, desmoglein, and PrP^c^ (Fig. 4C), while, as expected, desmoglein and E-cadherin did not interact.

**Table 1:** PrP^c^ partners in rafts.

Identified proteins	Accession n^o^.	*M*r	Peptide matches
Desmoglein 2	gi 416178	122	9
Plakophilin 2a	gi 1871541	93	4
Plakoglobin	gi 15080189	82	9
Desmoyokin	gi 627367	312	17
Desmoplakin	gi 3702136	332	38
gamma-actin	gi 17511847	42	8
beta-actin	gi 16359158	42	10
alpha2 Spectrin	gi 1805280	285	10
Annexin isoform 2	gi 16306978	39	18

Raft extracts from differentiated Caco-2/TC7 cells (10 days) were immunoprecipitated with anti PrP^c^ antibodies. The presence of PrP^c^ in the resulting material was checked by western blot before identification of the proteins interacting with PrP^c^ by liquid chromatography-tandem MS (MS/MS). The number of peptide matches that was obtained after trypsination for each protein as well as the accession number (NCBI) and the molecular weight (*M*r) are reported.

### PrP^c^ invalidation impairs the sub-cellular localization of junction-associated proteins and desmosome structure

**Figure 5:**
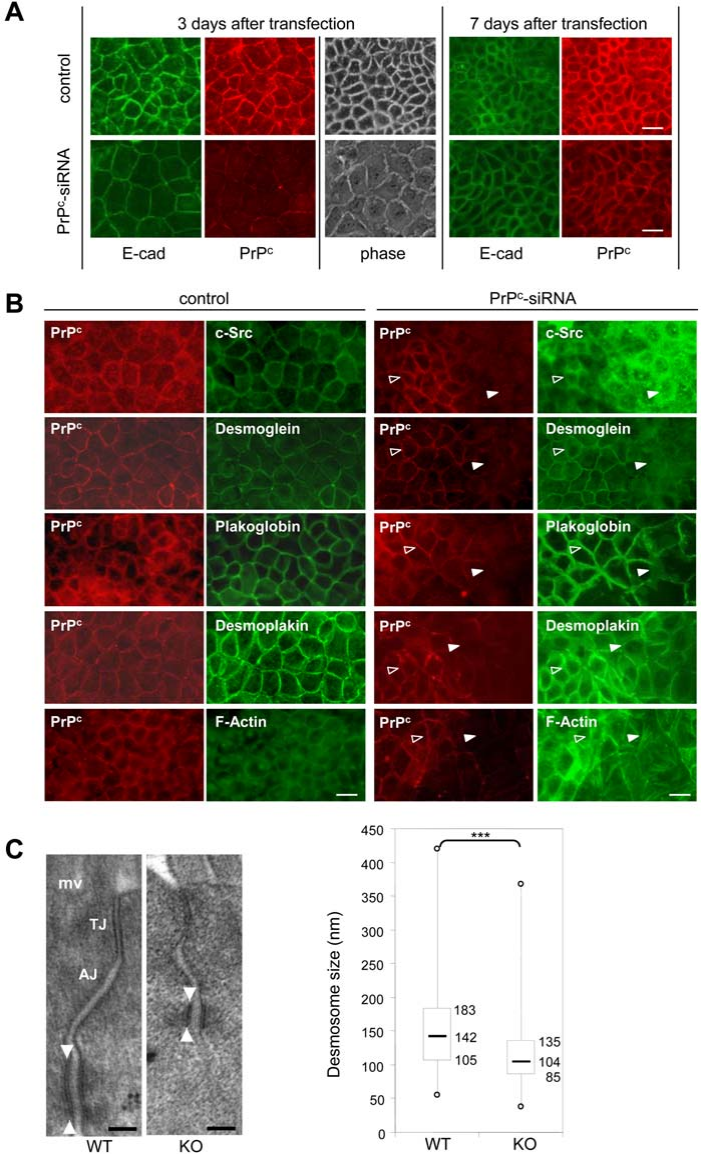
Effects of PrP^c^ invalidation on the sub-cellular localization of junctional PrP^c^ partners and on desmosome structure. (A): Caco-2/TC7 cells were transfected by PrP^c^-siRNA 3 days after seeding and the expression and localization of PrP^c^ (red, 12F10 antibody) and E-cadherin (green) were analyzed 3 days and 7 days after transfection in control (Oligofectamine or mouse PrP^c^-siRNA transfected cells) or human PrP^c^-siRNA transfected cells. Phase contrast image (phase) shows the morphology of the cell layer. (B): Immunofluorescence labeling of PrP^c^ (red), c-Src, desmoglein, plakoglobin, desmoplakin and F-actin (green) was performed in control cells (left panels) and in human PrP^c^-siRNA transfected cells 3 days after transfection (right panels). Control pictures are representative of the results obtained with scramble siRNA or with mouse PrP^c^ si-RNA or with cells incubated with the transfection agent, Oligofectamine. In human PrP^c^-siRNA transfected cells, fields combining zones where PrP^c^ was still present (open arrowhead) and zones where it was switched off (closed arrowhead) are shown. Note the difference in cell shape between both areas. Mouse 12F10 anti-PrP^c^ antibody was used for immunofluorescence labeling of PrP^c^ except for co-labeling with plakoglobin, in which rabbit polyclonal anti-PrP^c^ Ab703 antibodies were used. Bar: 20 µm. (C): Desmosomes were analyzed by electron microscopy in intestinal epithelium sections from PrP^c^ knock out (KO) and wild type (WT) mice. Note the electron-dense desmosomal plaque which was shorter in PrP^c^-knock-out than in wild type mice (arrowheads). mv: microvilli, TJ: tight junction, AJ: adherens junction. Bar: 100 nm. Quantification of desmosome size was represented in the lower graph. Box and whisker plots show median desmosomal length (horizontal line), 25th and 75th centiles (box), and range (whiskers) in the wild type (WT, n = 135, three mice) and PrP^c^-knock out mice (KO, n = 135, three mice), ***p<0.001.

Based on its interaction with desmosomal proteins, studies were undertaken to determine whether PrP^c^ could be involved in the organization of cell–cell junctions. Enterocytes were thus treated with human PrP^c^-siRNA, before the onset of cell polarity and the formation of cell–cell junctions. A kinetic analysis of PrP^c^ levels in transfected cells, by immunofluorescence, showed that in 50 to 60% of the cells the levels of the endogenous PrP^c^ were dramatically decreased 2 days (not shown) or 3 days after transfection (Fig. 5A) as compared to control cells, i. e. cells transfected with an inefficient mouse PrP^c^-siRNA or with a scramble siRNA or cells incubated with Oligofectamine only, and returned to the control levels from 4 days after transfection (not shown).

In a first attempt, the junctional state was assessed 3 and 7 days after transfection by the analysis of the expression and localization of E-cadherin, which does not interact with PrP^c^ but is the most studied marker of cell–cell junctions. Three days after transfection (i.e 6 days after seeding), junctional complexes were already formed in the center of expanding cell clusters. In cells where PrP^c^ expression was specifically impaired, the E-cadherin still localized to the lateral membranes of adjacent cells but its labeling intensity was decreased (Fig. 5A). Moreover, in the same fields, the cells appeared enlarged as compared with the three control conditions, as clearly noticeable in phase contrast picture (Fig. 5A). When the effect of human siRNAs on the expression of PrP^c^ was no longer observed (day 7, Fig. 5A), the size of the cells and the amount of the junctional E-cadherin were rescued. We then analyzed the expression and the sub-cellular localization of the junctional partners of PrP^c^ by immunofluorescence (Fig. 5B). To better compare cells that still expressed PrP^c^ (40–50%) or not (50–60%), fields that combined the two cell populations of human PrPc-siRNA transfected cells are shown (Fig 5B, right panels) along with pictures representative of the results obtained in the three control conditions (Fig. 5B, left panels). Cells exhibiting a net decrease of PrP^c^ levels were systematically enlarged. In these cells, the amount and/or the sub-cellular localization of the junctional partners of PrP^c^ were modified: c-Src was found essentially intracellular, desmoglein, plakoglobin and desmoplakin labelings were lowered, and numerous actin stress fibers could be visualized in large cells that no longer expressed PrP^c^.

The role of PrP^c^ on structural organization of cell–cell junctions was further analyzed in intestinal epithelium of PrP^c^ knock out mice. Ultrastructural analysis revealed drastic changes in the length of the desmosomal plaque (Fig. 5C upper panel), which was not compensated by their number (not shown). Quantification of the length of desmosomes indicated a distribution of their size concentrated between 83 to 134 nm in knock out mice instead of 100 to 183 nm in wild type mice (Fig. 5C lower panel).

### PrP^c^ invalidation impairs completion of cell division and results in a shortening of intestinal villi

**Figure 6:**
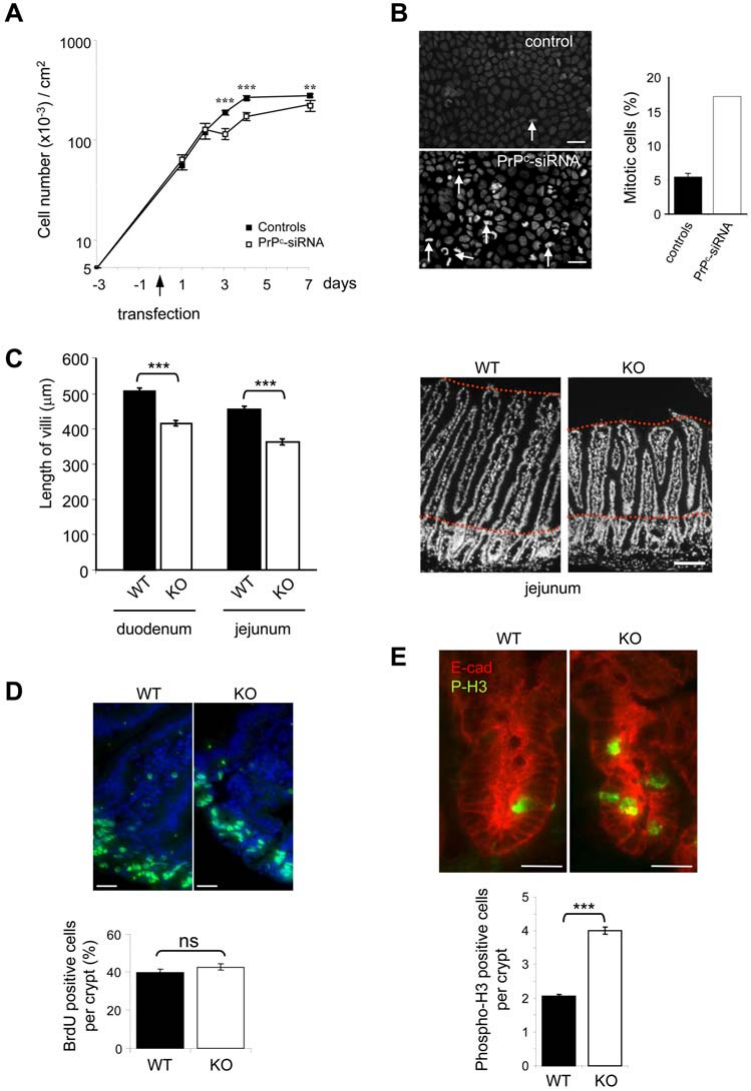
Effects of PrP^c^ invalidation on the completion of cell division and length of intestinal villi. (A): Caco-2/TC7cells were numbered all along the experiment from 3 days before transfection (−3) to 7 days after transfection. Controls combine results obtained in the three conditions described in figure 5 (scramble siRNA, mouse PrP^c^ si-RNA, Oligofectamine). PrP^c^-siRNA condition corresponds to the results obtained after specific human PrP^c^-siRNA transfection. Results are from 4 independent experiments. **p<0.01, ***p<0.001. (B): In the same experiments, cells that showed nuclear division phases were counted after staining with DAPI. In the left panels arrows point examples of metaphases or telophases that were counted. In each condition, 1000 cells were analyzed and the percentage of mitotic cells in controls (black bar, same three conditions as above) or in human PrP^c^-siRNA (white bar) transfected cells is reported in the right panel. Error bar shown in histogram of controls represents the statistical analyses performed when combining the three control conditions. C: sections of duodenum and jejunum fragments from wild type (WT) and PrP^c^ knock out (KO) mice (2 mice in each group) were stained with DAPI (right panel, bar: 100 µm). Sixty villi were measured for each mouse and fragment. No difference was observed between the 2 mice of each group (not shown), but significant differences (***p<0.001) were obtained between wild type (black bars) and knock out mice (white bars) in both duodenum and jejunum fragments. (D): After 1.5 hour BrdU intraperitoneal injection, sections from the jejunum fragment of wild type (WT) and knock out (KO) mice were performed. Nuclei were visualized with DAPI staining (blue). BrdU-stained proliferating cells (green) are limited to the crypts in wild type and knock out animals. Bars: 10 µm. Knock out mice display similar number of positive BrdU cells per crypt as compared with wild type mice (quantification in the right panel graph). No significant difference was revealed by statistical analysis (ns, student's *t* test). (E): Immunofluorescence analyses of phospho H3 (green) and E-cadherin (red) were performed on intestine sections from wild type (WT) or PrP^c^ knock out (KO) mice (3 mice in each group). Pictures of crypt staining are shown (upper panels; bars: 10 µm) and the number of phospho-H3 positive cells per crypt is reported (155 crypts were counted in each group). ***p<0.001.

When compared to the three control conditions, we noticed an enlargement of human PrP^c^ siRNA-treated dividing enterocytes (Fig. 5A) that could reflect the impact of PrP^c^-expression on cell proliferation. Analysis of cell growth (Fig. 6A) showed an arrest of the increase in cell numbers between 2 and 3 days after transfection, i.e during the period when PrP^c^ expression was significantly decreased by human siRNAs. Surprisingly, DAPI staining of the nuclei showed that growth arrest was paralleled with a net increase of the number of mitosis, without apoptosis, in cells that did not express PrP^c^ anymore (Fig. 6B). In parallel, the morphological examination of the intestinal epithelium of PrP^c^ knock out mice revealed a net decrease in the length of the villi in both duodenum and jejunum segments in comparison with their wild type counterparts (Fig. 6C). To analyze the impact of PrP^c^ expression on cell proliferation within the intestinal epithelium, PrP^c^ knock out and wild type mice were pulse-labeled with BrdU for 1.5 hour prior to sacrifice. No significant difference in the number of BrdU-labeled cells in sections of jejunal crypts was observed between wild type and knock out mice (Fig. 6D), suggesting that S phase was not affected. By contrast, labeling of the mitosis-associated phospho-H3 revealed a net increase of mitotic cells in the crypt compartments of intestine from PrP^c^ knock out mice as compared with wild type mice, while no obvious change in the size of the crypts was observed.

## Discussion

Our present results demonstrate that the cellular prion protein, PrP^c^, exhibits a dual distribution between the nucleus, in actively dividing cells, and cell–cell adhesion sites in polarized/differentiated cells. Interestingly, the membrane PrP^c^ interacts with desmosomal proteins as well as with actin and actin-binding proteins at cell–cell junctions. Furthermore, we show that down regulation of PrP^c^ expression in Caco-2/TC7 enterocytes lead to a complex pattern of alterations in both cell architecture and completion of the cell division process. These results are strengthened by the analysis of the intestinal epithelium of PrP^c^ knock out mice, in which intestinal villi were found shortened and the size of enterocyte desmosomes decreased as compared to wild type mice.

Contrary to abnormal prion proteins [36]–[38], a targeting of the normal PrP^c^ isoform to the nucleus has been rarely reported, [39], [40]. We demonstrate here that in intestinal epithelial cells, such a nuclear targeting is observed only in actively dividing cells, both in cultured enterocytes and in the intestinal epithelium *in vivo*. The characterization of the nuclear pattern of PrP^c^ isoforms revealed that it is similar to that of membrane-associated PrP^c^ in terms of glycosylation and presence of a GPI anchor. Analysis of protein stability shows a much longer half-life of PrP^c^ in the nucleus than in plasma membrane, where junctional proteins are rapidly recycled. Nuclear PrP^c^ stability could rely on the association with particular sub-nuclear compartments, such as PML bodies [41], a localization compatible with the pattern of nuclear PrP^c^ staining that we observed. Contrasting with the misfolded protein [38], we show an exclusion of normal PrP^c^ from the nucleolus. Altogether, our results asked the question of its biological role in the nucleus of dividing cells. Upon PrP^c^ down regulation in cultured enterocytes, we observed modifications of cell morphology and an arrest of cell growth. This observation was consistent with the decreased villus size of the intestinal epithelium that we observed in PrP^c^ null mice. In several mouse models, it has been shown that reduced intestinal crypt cell proliferation is associated with shorter villi [42], [43]. The growth arrest observed in PrP^c^ siRNA-transfected cells was paralleled by an increase of mitotic cells. Further analysis of cell cycle perturbations was rendered difficult by the moderate transfection efficiency of Caco-2/TC7 cells by siRNA (50%). Nevertheless, the absence of S phase overt perturbation in crypts from PrP^c^ null mice, as shown by BrdU incorporation experiments, along with the important increase of the number of mitotic cells in the crypt compartment suggest that PrP^c^ invalidation would affect the mitosis process. PrP^c^ has been shown to associate with tubulin [44] [45]. In addition, desmoplakin, that we identified as a PrP^c^ partner, participates to the organization of microtubules in keratinocytes, through the recruitment at cell–cell junctions of a centrosomal protein, which is required for microtubule anchoring [46]. However, it cannot be concluded yet whether all the phases of mitosis or more particularly the last step of cytokinesis are slowed down through PrP^c^ invalidation.

Another important finding of our study is the identification of desmosomal proteins as PrP^c^ partners at cell–cell junctions. We had already identified c-Src kinase as a partner of PrP^c^ in polarized enterocytes and demonstrated that PrP^c^ does not interact with E-cadherin [24], asking the question of the partner that could link PrP^c^, which is anchored in the outer leaflet of the lateral membrane, to Src, which is localized in the inner one. Our data are consistent with the existence of a molecular complex in which the transmembrane desmosomal cadherin desmoglein is this link between PrP^c^ and c-Src. Involvement of PrP^c^ in the regulation of desmosome organization, and more generally in cell architecture, is further supported by our demonstration of alterations in the junctional targeting of Src and desmosomal proteins upon PrP^c^ down-regulation by siRNA during the establishment of enterocyte cell–cell contacts. Importantly, absence of PrP^c^ *in vivo* results in alterations in the size of desmosomes in intestinal epithelial cells. This structural perturbation could reflect modifications of assembly and/or stability of the desmosomal-protein complex in the absence of PrP^c^, as described for invalidation or mutation of other desmosome-associated proteins, such as desmoglein, plakoglobin or plakophilin families, in murine models or human pathologies (for review, see [47]). Desmosomal proteins have emerged as adhesion molecules that not only play critical structural roles but are also involved in signaling pathways [48]. Our data further support the hypothesis of the involvement of PrP^c^, in association with its desmosomal partners, in a signaling platform of the junctional state.

In conclusion, our results indicate that the normal cellular prion isoform PrP^c^, by its differential nuclear or junctional localization, must be considered as a potential actor of the balance between proliferation and polarization/differentiation of epithelial cells, through interaction with c-Src and with desmosome- and cytoskeleton-associated proteins. As such PrP^c^ could be involved in the homeostasis of renewing epithelia.
