# Correction: The Cellular Prion Protein PrP^c^ Is Involved in the Proliferation of Epithelial Cells and in the Distribution of Junction-Associated Proteins

**DOI:** 10.1371/annotation/e5a42567-afa3-422a-886e-44ca642c6fe2

**Published:** 2008-09-25

**Authors:** Etienne Morel, Stéphane Fouquet, Carine Strup-Perrot, Cathy Pichol Thievend, Constance Petit, Damarys Loew, Anne-Marie Faussat, Lucile Yvernault, Martine Pinçon-Raymond, Jean Chambaz, Monique Rousset, Sophie Thenet, Caroline Clair

The fourth author's name appears incorrectly in the citation. The correct citation should read: Morel E, Fouquet S, Strup-Perrot C, Pichol Thievend C, Petit C, et al. (2008) The Cellular Prion Protein PrPc Is Involved in the Proliferation of Epithelial Cells and in the Distribution of Junction-Associated Proteins. PLoS ONE 3(8): e3000. doi:10.1371/journal.pone.0003000

