# Correction: The Cellular Prion Protein PrP^c^ Is Involved in the Proliferation of Epithelial Cells and in the Distribution of Junction-Associated Proteins

**DOI:** 10.1371/annotation/0b364095-9f93-4cb9-9a2e-aae5ed1bf362

**Published:** 2008-09-25

**Authors:** Etienne Morel, Stéphane Fouquet, Carine Strup-Perrot, Cathy Pichol Thievend, Constance Petit, Damarys Loew, Anne-Marie Faussat, Lucile Yvernault, Martine Pinçon-Raymond, Jean Chambaz, Monique Rousset, Sophie Thenet, Caroline Clair

There was an error in the affiliations of the tenth and twelfth authors, and in Affiliation 4. The correction affiliations should appear as shown here:

Etienne Morel^1,2,3^, Stéphane Fouquet^1,2,3^, Carine Strup-Perrot^4^, Cathy Pichol Thievend^1,2,3^, Constance Petit^1,2,3^, Damarys Loew^5^, Anne-Marie Faussat^1,2,3^, Lucile Yvernault^1,2,3^, Martine Pinçon-Raymond^1,2,3^, Jean Chambaz^1,2,3,6^, Monique Rousset^1,2,3^, Sophie Thenet^1,2,3,6^, Caroline Clair^1,2,3^

**1** Centre de Recherche des Cordeliers, Université Pierre et Marie Curie-Paris 6, UMR S 872, Paris, F-75006 France, **2** INSERM, U 872, Paris, F-75006 France, **3** Université Paris Descartes-Paris 5, UMR S 872, Paris, F-75006 France, **4** UPRES EA 27-10, IRSN, IGR, LRPAT, Villejuif, F-94805 France, **5** Laboratoire de Spectrométrie de Masse Protéomique, Institut Curie, Pavillon Pasteur, Paris, F-75248 France, **6** Ecole Pratique des Hautes Etudes, Laboratoire de Pharmacologie Cellulaire et Moléculaire, Paris, F-75006 France

